# Intensionality is more cognitively demanding than false belief in adults: evidence from a non-inferential sentence-reading task

**DOI:** 10.1038/s41598-024-51181-w

**Published:** 2024-01-04

**Authors:** Elaine Kit Ling Yeung, Him Cheung

**Affiliations:** 1 School of Psychology, University of Birmingham, Birmingham, UK; 2 Department of Psychology, The Education University of Hong Kong, Hong Kong, China

**Keywords:** Psychology, Human behaviour

## Abstract

Children are said to understand false belief if they can appreciate an agent’s wrong description of an object as a result of misinformation, and intensionality if they can appreciate and switch between alternative descriptions from different epistemic viewpoints. Most previous studies have investigated the developmental trajectories of these capacities in the age range from 3 to 10 years aiming to discern their conceptual nature. The present research examines whether intensionality incurs lower performance accuracies and longer response times than false belief in adults, using a task in which participants read sentences that explicitly state an agent’s beliefs. Experiment 1 showed that participants were less accurate in rejecting verbal probes that contradicted an agent’s alternative than false thoughts about objects. Experiments 2 and 3 replicated this finding using thoughts about object identities but not properties. These results suggest that compared to false belief, intensionality is cognitively demanding for adults to process because of the availability of more than one identity candidate under the agent’s perspective.

## Introduction

The mind is metaphorically opaque as it may not always represent reality faithfully and completely. Different agents may hold different representations of reality depending on a host of epistemic and motivational factors, and thus the contents of such representations need to be understood relative to the respective agents, not reality. Opacity of mind is central to developing a mature theory of mind (ToM)^1–3^ which refers to the ability to reason about mental states and to use them to explain and predict behaviour^4,5^. A full-fledge understanding of the concept of belief in ToM entails opacity understanding in two forms^1,6^. First, an agent may hold a *false belief* because of misinformation or a lack of access to true information, i.e., the agent’s mind is opaque to reality^7^. Second, an agent may recognise only some, not all, of the alternative descriptions of reality, i.e., the agent’s mind is opaque to some aspects of reality, as in the classic *intensionality* (or *aspectuality*) problem in which one could intend for Venus to be the morning star but not the evening star, or vice-versa^2^.

While some authors have shown that children pass false belief well before they pass intensionality and thus argue for a fundamental conceptual difference between the two capacities^2,6,8,9^, others have counter-argued that observed performance differences could be attributed to some pragmatic factors and that false belief and intensionality are actually more conceptually unified under ToM than often assumed^10^. These extraneous pragmatic factors confuse children within the age range from 4 to 9 years especially in true belief tasks (i.e., tasks in which the agent has full information about the target object or event), which are often used as a baseline to which false belief and intensionality tasks are compared^11–13^. In the present study, therefore, we assumed conceptual equivalence between false belief and intensionality and compared them on performance accuracy and response time in adults. A well-designed experimental task minimising procedural differences between the conditions was used. If intensionality incurs lower accuracies and/or longer response times than false belief in adults, such differences may only be attributed to differences in processing cost or mental effort, not conceptual mastery because of the conceptual equivalence assumption and adults’ supposed full-fledged understanding of ToM. A question to follow is why intensionality may incur greater processing cost than false belief. Hence, we attempt two questions in the present study. First, does intensionality incur greater processing cost than false belief? Second, why does intensionality incur greater processing cost than false belief?

### False belief versus intensionality

The false belief test, which serves as the conventional benchmark for ToM development in children, assesses the understanding that mental representations can be completely opaque to reality^4,14^. For instance, in the unexpected transfer paradigm, the child predicts where an agent will look for an object that has been transferred to a new location without the agent knowing the transfer^5^. In the appearance-reality paradigm, the true identity of an object with a deceiving appearance is revealed to the child, who is then asked to predict what another person will think the object is just by looking at it^4,15^. Both paradigms examine the child’s understanding that other agents could hold beliefs that are in conflict with reality because of insufficient information. Results have shown that children typically pass false belief using these traditional paradigms at the age of 4^16,17^. Nevertheless, it has been pointed out that passing of false belief does not indicate full understanding of opacity because false belief tests typically do not capture intensionality^1–3^.

The problem of intensionality occurs when two expressions that refer to the same object, or referent, may not be interchanged when describing the mental content of an agent. For instance, Venus is known as both the evening star and the morning star, but knowing that Venus is the evening star does not entail knowledge of it being the morning star. Hence, in the sentence “John believes that he has just seen the morning star”, “morning star” cannot be replaced by “evening star” if John does not know that “evening star” is an alternative description of “morning star”, i.e., John’s mind is opaque to the “evening star” description of the same referent Venus. Compare this to following sentence in which “morning star” and “evening star” are interchangeable because no reference to John’s belief is made: “John has just seen the morning/evening star”.

Intensionality, or the understanding that the mind can be opaque to some alternative descriptions of reality, could be assessed using objects having dual identities describable with co-referential expressions. Some previous studies have shown that children do not fully grasp intensionality until 6 or 7 years^6,8,9,18–21^. According to these authors, younger children typically find it difficult to reject statements in which the agent uses an alternative expression that is epistemically unavailable to him or her to refer to a dual-identity object, compared to traditional false belief. These authors thus maintain that intensionality and false belief are distinct conceptual capacities having different developmental trajectories. On the other hand, Rakoczy et al.^10^ considered the effects of extraneous pragmatic factors in some often used false belief and intensionality tasks, and compared 3- to 6-year-olds’ performances on false belief and a simplified intensionality test in which the effects of these extraneous factors were minimised. In this test, the experimenter first showed the child and an agent (i.e. a puppet) one aspect of a dual-identity object, such as showing that a pen-rattle works as a pen (without shaking it to make a rattling sound), and placed it in location 1. The experimenter then took the puppet away and showed the child that the object in location 1 was not only a pen but also a rattle (shaking it). After the puppet returned, the experimenter covered the pen-rattle with her hands, called it a rattle (shaking it) and transferred it to location 2. Finally, the experimenter asked the test question, “Where will the puppet look for the pen?” The authors claimed that the task captured intensionality because it required the child to hold in mind that the puppet did not know that the pen was also the rattle now placed in location 2. Results showed that intensionality measured with this task was no more difficult than and correlated highly with false belief. The findings were replicated in several other studies using the same intensionality paradigm^11–13^. Rakoczy et al. thus concluded that intensionality and false belief are conceptually more similar than different and that they represent different aspects of a unified ToM capacity. The debate surrounding conceptual equivalence between intensionality and false belief primarily concerns conceptual mastery and development in children and hence may not be directly relevant to the present focus on processing cost in adults.

### The mental files theory

Perner et al.^19^ adopt the mental files theory to describe the mental operations involved in false belief and intensionality. In this theory, different mental operations are assumed for false belief versus intensionality and therefore different processing costs may be expected for the two capacities. A mental file is a cognitive structure used to track an individual’s knowledge or belief about an object under a certain label^19,22–24^. Each distinctly labelled file corresponds to a distinct conceptual perspective on a referent; a new file needs to be opened when the referent is labelled in another way. Each file stores predicate information (e.g. object property, location) about the concept behind the file label. Hence, the information content of a file is determined by the label, not the referent.

**Figure 1:**
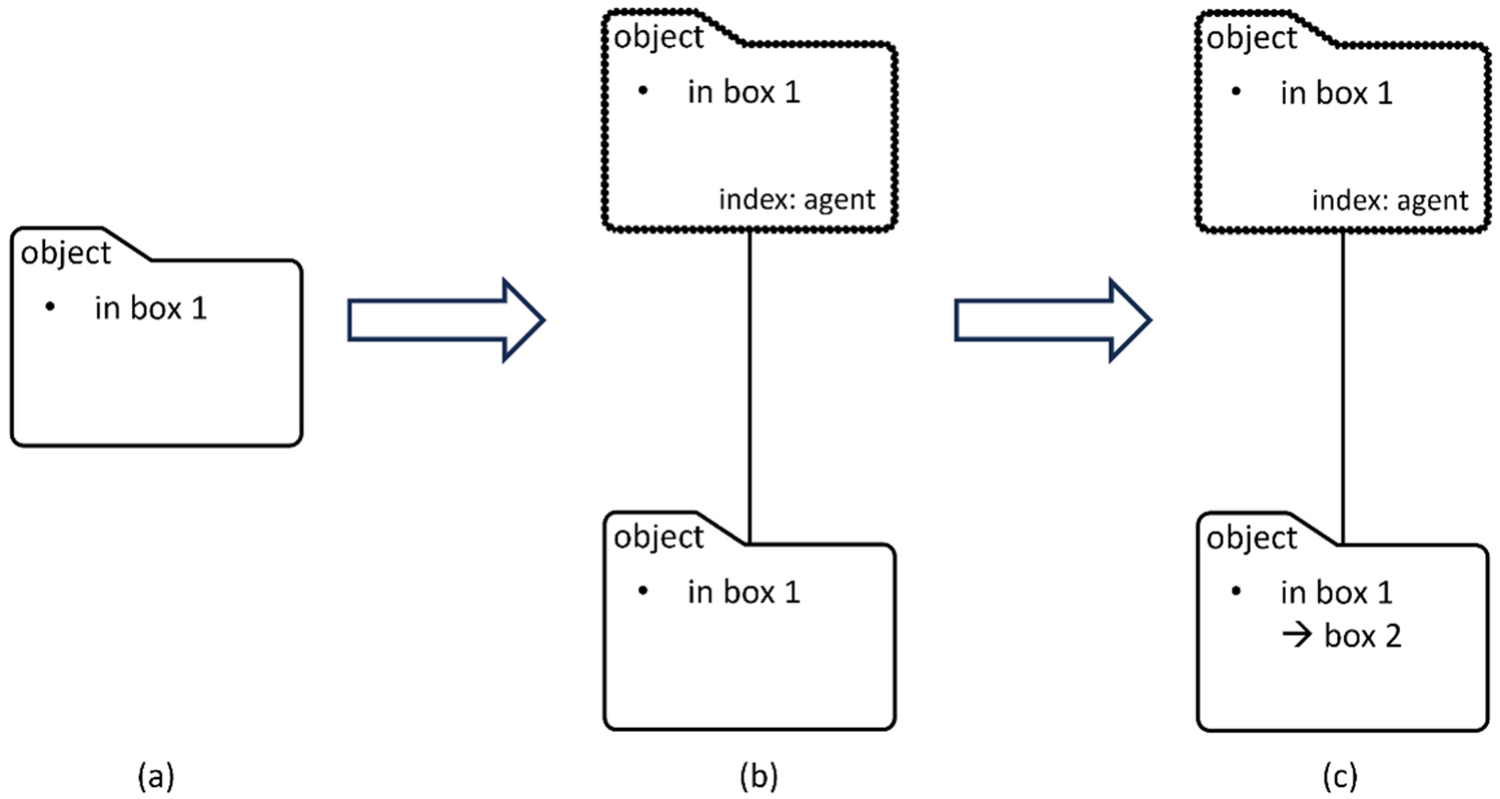
How mental files track an agent’s false belief about object location. (**a**) The observer creates a regular file to track her own belief about object location (box 1); (**b**) the regular file (bottom) is copied to a vicarious file (top) to track an agent’s belief about object location before object transfer; (**c**) information in the vicarious file does not synchronise with the location update in the regular file after object transfer from box 1 to box 2, which is unknown to the agent.

In the mental files theory, “regular files” contain an observer’s thoughts about a referent whereas “vicarious files” are usually copies of regular files describing the observer’s representation of another agent’s thoughts about the same referent. For instance, the observer sees a dog (the referent) and a regular file under the label “dog” containing knowledge about the dog (e.g. that it is black) is created. This file is copied to a vicarious “dog” file if the observer thinks another agent also sees the referent as a dog. A vicarious file can also be created and deployed without a regular file under the same label^18,19^. For example, the observer reckons that another agent has mistaken the dog for a wolf for some reason and a vicarious “wolf” file is created to represent the agent’s wrong thought about the referent. Files tracking the same referent, i.e., co-referential files, are said to be “linked”. Linkage between co-referential but distinctly labelled files indexed to the same agent allows information to freely flow between them. However, when linking a regular file and its corresponding vicarious files, the vicarious files may not synchronise with updates in the corresponding regular file if the agent is thought to have no access to that updated information: This is the key to understanding false beliefs about object property or location (Fig. 1).

**Figure 2:**
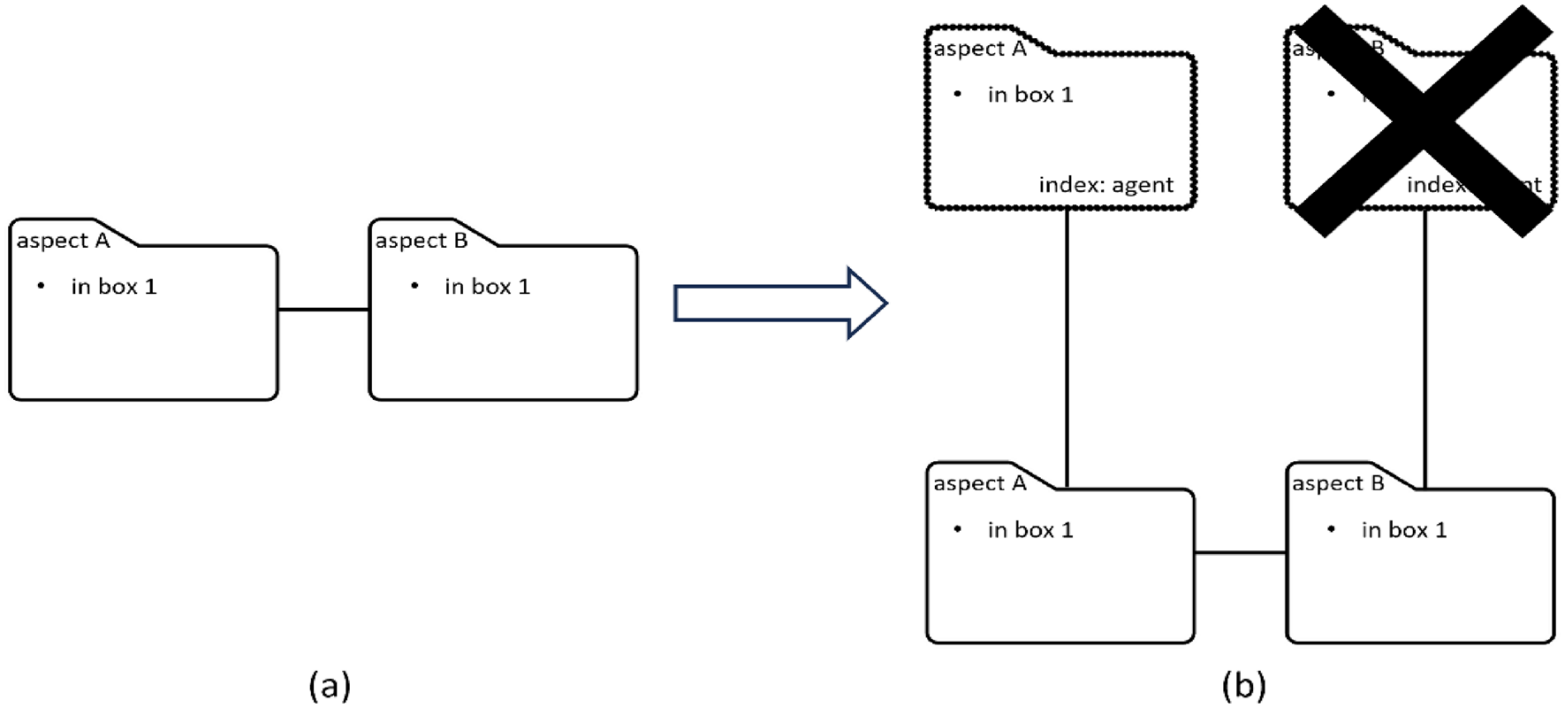
How mental files track an agent’s belief in a dual-identity intensionality situation. (**a**) The observer creates two regular files to track her own belief about the two respective identities, i.e., aspects A and B, of a dual-identity object. The link between the two files indicates that they are co-referential. (**b**) Vicarious files (top) are created by copying the corresponding regular files. The vicarious file representing aspect B is then deleted to indicate that this identity of the object is unknown to the agent.

In a dual-identity intensionality situation, the observer holds two linked regular files to represent the respective identities of the referent (Fig. 2). Yet she knows that only one of the two identities is known to the tracked agent and thus the vicarious file corresponding to the unknown identity has to be deleted to represent the agent’s point of view. The observer needs to engage in mental operations resulting in only one out of two vicarious files being linked to its corresponding regular file. When considering false belief about object identity, in contrast, the observer does not need to decide between multiple vicarious files because there is only one, representing a false identity from the agent’s point of view. Hence, in the case of identity false belief, the observer needs to make no further decision on alternative vicarious files after distinguishing the agent’s point of view from her own.

### The present study

According to the mental files theory, false belief and dual-identity intensionality do not entail the same mental operations. The assumed difference in mental operations implies that these two aspects of ToM may incur different online processing costs. Specifically, while the observer needs to decide which one of two vicarious files to delete when considering the agent’s perspective in a dual-identity intensionality situation, there is no need to decide between multiple vicarious files when thinking about identity false beliefs. Given this background, the present study compares false belief and intensionality reasoning in adults to address two questions, with a focus on non-inferential processes in ToM. First, does processing beliefs involving dual-identity intensionality incur higher processing cost than processing false beliefs about identity? Second, does intensionality incur higher processing cost than false belief only when it involves alternative identity descriptions of a dual-identity object, not alternative descriptions of properties under one identity? These questions were addressed in three experiments. Experiment 1 compared participants’ accuracies and response times in rejecting probes that contradicted an agent’s false versus alternative thoughts about object identities. Experiments 2 and 3 examined whether the pattern of results from Experiment 1 could be extended to thoughts about object properties.

Past research on beliefs involving intensionality has been focused on identifying the timepoint at which children fully grasp the concept of belief. However, this approach provides limited insight into the cognitive mechanisms involved in processing beliefs involving intensionality, or more generally, the processing of ToM. A cognitive approach teases apart three key processes in ToM: inferencing, storage, and use of information about mental states; the processes are separate, and one might engage in some but not all three processes while performing ToM activities^14,25^. Hence, despite that many ToM paradigms do require participants to infer the mental states of agents, inferencing itself is not a necessary component in ToM activities. Pertaining to intensionality in beliefs, inferring an agent’s belief is not always necessary as demonstrated in the classic philosophical example where someone knows Venus as “the morning star” but not as “the evening star”. Despite that these two terms convey different information about Venus, understanding why the person only knows Venus as “the morning star” is not required to recognise she cannot think of Venus as “the evening star”.

Drawing from empirical evidence, studies have shown that difficulties in ToM processing still occur in children^26–29^ and adults^26,30–33^, even when no inferencing is required; particularly, comparable results to inferential ToM tasks were observed even in studies that did not set up stories but rather just presented direct descriptions of agents’ beliefs, both in children and adults^26,29,30^. These findings suggest that ToM processing is demanding not only in making mental state inferences but also in holding relevant information in mind and/or acting upon it. Neuroscience findings corroborate behavioural findings showing activation of the same brain regions when one processes belief information, regardless of whether inferencing is required^34,35^, suggesting overlapping mechanisms in belief processing both with and without the need for inferences. Distinguishing the three processes in studying ToM activities also offers a way to elucidate the cognitive basis of the separate processes involved in ToM, rendering non-inferential ToM processing a meaningful topic of study.

Therefore, in the current study, we adapted the non-inferential paradigm developed by Apperly et al.^30^ to study adults’ processing of false belief and intensionality without engaging in belief inferencing. More specifically, we focused on adults’ remembering of false belief and beliefs involving intensionality. In the original paradigm by Apperly et al.^30^, sentences that described the reality about an object and the belief held by the agent about the same object or another object were presented in each trial; each trial always presented only one belief of the agent, which matched or mismatched, or was unrelated to the reality information about the target object. Following the procedure of this paradigm, the agent’s beliefs were also explicitly stated in sentences in the current study; participants did not have to make inferences about what the agent believed. Participants were required to reject a depiction of the agent’s belief featuring a true aspect of the target object unavailable from the agent’s perspective in both the intensionality condition and the false belief condition. It was because only an alternative true aspect was available from the agent’s perspective in the intensionality condition, and the agent’s belief was not true about the object in the false belief condition. As will be further elaborated, the use of a non-inferential task also allows for more closely matched experimental conditions and a wider range of objects suitable for being used as stimuli. Thus, this approach is advantageous for studying nuanced effects in the cognitive processes of adults with the use of multiple trials, compared to traditional developmental paradigms involving belief inferences that aim to study conceptual development in children, where the use of minimal (usually one to four, and typically two) items is considered adequate.

## Experiment 1

Experiment 1 compared adults’ performance accuracies and response times when processing beliefs involving intensionality versus false beliefs regarding object identity, to test whether processing the former incurs higher cost than processing the latter. Lower accuracies and longer response times indicate higher processing cost.

### Methods

#### Participants

Power was calculated by simulation using R 4.1.1^36^ with the package SIMR^37^. Results showed that test of fixed effect in a multilevel logistic regression model specified with random intercepts with a sample size of 35 reached 79.90% power, 95% CI [77.28%, 82.34%]. The simulation model assumed a small effect size (odds ratio 1.68)^38^ and a random intercept variance of 1. Thirty-five Cantonese–Chinese-speaking, right-handed participants (19 females) aged 18 to 35 years (*M* = 21.57 years, *SD* = 3.53 years) were recruited from the Chinese University of Hong Kong for course credit or a small incentive of approximately USD6.4. They had normal hearing and normal or corrected-to-normal vision. None reported any cognitive or language impairment. Ethical approval was obtained from the Survey and Behavioral Research Ethics Committee, Social Sciences Panel of the Chinese University of Hong Kong. All procedures and methods were performed in strict accordance with the guidelines and regulations set forth by the Committee. All participants gave informed consent before participation.

#### Design and stimuli

A one-way within-subject design was used with belief type (intensionality (IN) vs. false belief (FB)) as the independent factor and response accuracy and response time as the dependent measures. Each condition included 10 trials, totalling 20 test trials, all requiring the participants to reject a picture probe, the last of a series of four slides in each trial, as an accurate depiction of an agent’s belief with reference to the three previous slides which provided the background story information. In addition, 80 filler trials were included to distract the participants and to discourage them from adopting response strategies. There were 8 types of fillers, each having 10 trials. Thirty of these fillers required a “no” response while the remaining 50 required a “yes” response. Thirty fillers required the participants to consider the agent’s thoughts, i.e., making decisions on picture probes depicting what the agent thinks an object is (belief probes); 50 fillers required them to consider the story reality, i.e., making decisions on picture probes depicting what an object really is (reality probes). Therefore, both test (20) and filler trials (80) put together, half (50) required a “yes” response while the other half a “no” response; half used belief probes while the other half reality, non-mental probes.

Ten sets of three nouns, presented in text form, were used in the 20 test trials. In each set, two of the three nouns were alternative descriptions of a dual-identity object. For example, for an object described as both a fork and a spoon, “fork” (N1) and “spoon” (N2) were the alternative descriptions. The third noun (N3) was a false description, “knife” in this case. In a test trial, slides 1 and 2 included N1 (“The object on the chair is a fork”) and N2 (“It is also a spoon”) to reveal the two alternative identities in a counterbalanced order. The content of slide 3 depended on belief type. In an IN trial, slide 3 revealed the one alternative identity that the agent knows, i.e., N1 (“He thinks the object on the chair is a fork”); in contrast, slide 3 in an FB trial revealed a false belief using N3 (“He thinks the object on the chair is a knife”). Slide 4 showed the same picture probe across the two conditions, depicting the agent’s thought with the identity that is unknown to him or her, i.e., N2, (“spoon” in this case) in a thought bubble. Participants were to decide on the probe’s veracity as quickly as possible. Other than the stimulus set for practice trials, each stimulus set was presented only once in each experimental block, each time in a unique condition.

We specified in the instruction that the agent happens to hold only one belief about object identity in each independent trial, regardless of trial type. In other words, the participant assumed that the agent held a belief about only one specific identity of the object not because she made such an inference from the story but because she was explicitly instructed to assume so. This premise may appear artificial at first glance, but it actually mirrors common daily life experiences in which we often possess knowledge about one aspect while lacking it in another (e.g. knowing the person living next door to be Mr. Smith without knowing he is also a firefighter), or hold a mistaken belief (e.g. thinking that it is Mr. Greens who lives next door), about an object or a person. In a non-inferential paradigm, we simply provided explicit information about this common limitation of one’s beliefs to the participants, without requiring them to make inferences from vignettes. Analogous examples in daily life, where we are told about someone else’s beliefs, are common. For example, Mrs. Smith’s daughter, Anna, who lives next door, might tell us her classmates only see Mrs. Smith just as their teacher but not the mother of one of their classmates, and plead with us to keep it a secret lest others feel they are being treated unfairly. In this case, the reason behind her classmates’ unawareness of Mrs. Smith being Anna’s mother is irrelevant.

The instructions applied to all types of trials, including both experimental trial types; nothing about the truthfulness of the belief was implied as the belief that the agent happened to hold in each trial could be either true or false. Therefore, it would not appear to the participant that the agent also knows that the object is a “spoon” in either the IN or FB condition; a negative response was hence expected in either condition because “spoon” was epistemically unavailable to the agent given his or her false belief (“knife”) in the FB condition and alternative belief (“fork”) in the IN condition, creating matched task demand across the two conditions. Semantic association between N2 and N1 and that between N2 and N3 were rated as similar by a separate sample of 43 participants in a pilot test. Internal validity was hence effectively maintained: the two experimental conditions were closely matched as the stimuli had comparable semantic distance from the probe, were highly similar in their form of presentation, the response probes were always identical, and both trial types uniformly demanded the same correct response. If intensionality is more demanding to process than false belief, it would be more difficult for the participants to reject “spoon” in IN than FB, for such rejections necessitated computation of intensionality and false belief in the respective conditions; rejection decisions could not be made on the basis of semantic distance between the to-be-rejected thought (N2) and the agent’s beliefs (N1 and N3). Another set of exemplar items include “mouse” (N1), “scanner” (N2), and “keyboard” (N3). All instruction and stimuli were in Chinese.

#### Procedure

Written informed consent was obtained online before the experiment. Participants first read a short briefing passage on the prevalence of dual-identity objects in daily life with two examples: a tomato, which could be a fruit and a vegetable, and a scarf, which could be used both as a scarf and a shawl. Participants were then told that in each trial they would read short sentences on the computer screen about the two identities of a dual-identity object, which is put either on a table or a chair in a room (slides 1 and 2). A sentence would follow describing an agent’s belief about or pointing at an object in one of the two locations (slide 3). It was stressed that in each trial the agent happens to hold only one belief about the target object. Hence, in the fork-spoon case, if the “fork” identity is known to the agent, the “spoon” identity must be unavailable, and the agent will not falsely believe that the object is a “knife”; if the agent falsely believes the object to be a “knife”, neither true identity (“fork” or “spoon”) is available from the agent’s perspective. In cases where the sentence merely describes the agent pointing at an object (in some filler trials), it does not imply what the agent believes the object to be. Finally, participants were to make a yes–no decision on slide 4 which was a picture probe depicting either what the agent thinks the object is (in the test and some filler trials) or what the object actually is (in some filler trials), using information from the three previous slides. Accuracies and response times were recorded.

**Figure 3:**
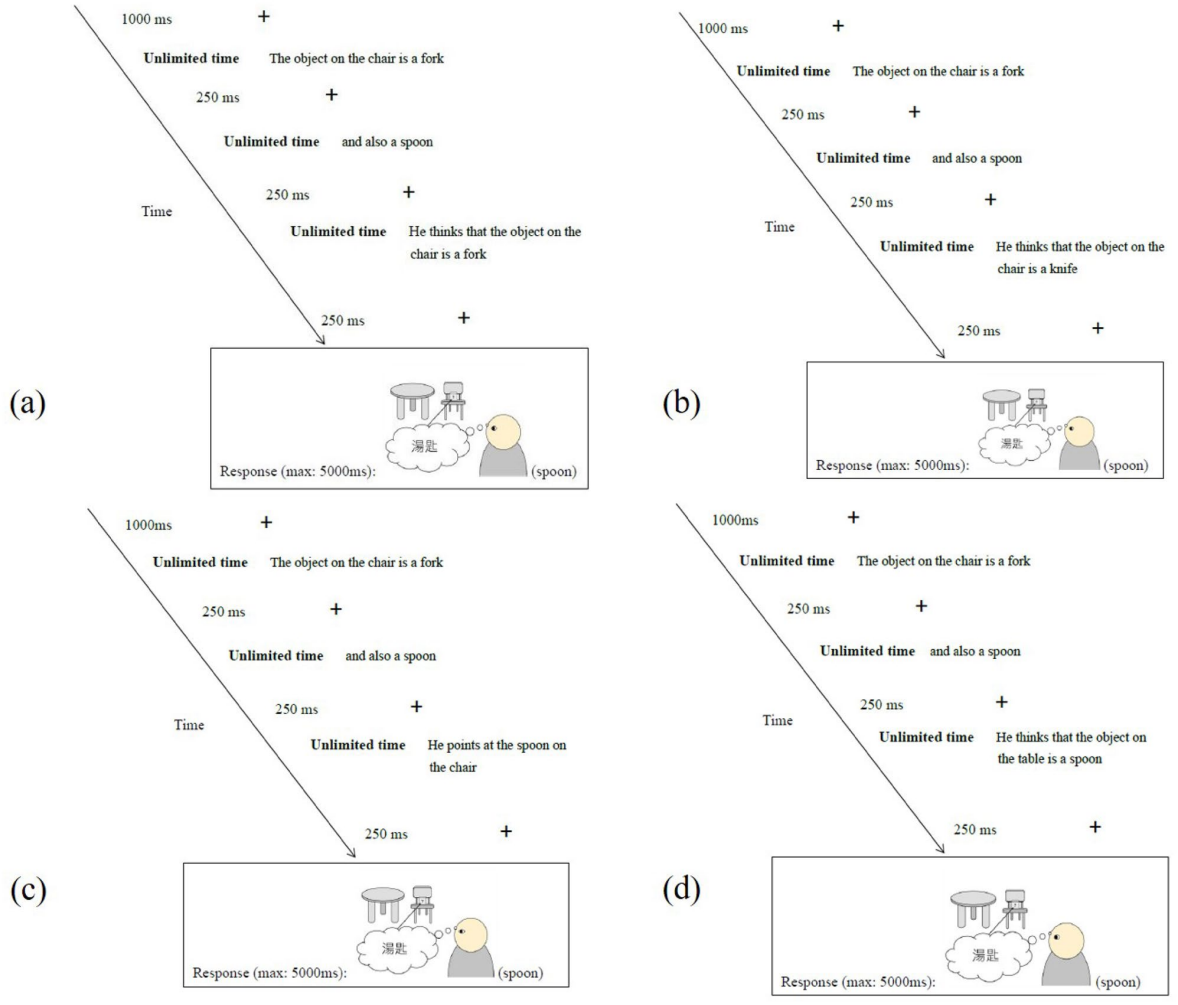
Exemplars of slides in experiment 1. (**a–d**) Depict the flow of an intensionality trial, a false-belief trial, a non-mental-verb filler and an incongruent-location filler respectively.

In each trial, a fixation cross appeared at the centre of the screen for 1000 ms and then slides 1 to 3 were presented in succession (self-paced), separated by a 250-ms fixation cross. Another 250-ms fixation appeared before slide 4 (picture probe), upon which participants made a yes–no response by pressing designated keys on the keyboard. See Fig. 3 for trial structure. Ten practice trials with feedback were used before the experimental trials; trial presentation was randomised. Presentation order of N1 and N2 in the test trials was counterbalanced. All stimuli were presented through the Gorilla Experiment Builder (www.gorilla.sc)^39^.

### Results and discussion

Results were pre-screened before analysis. Trials with response times shorter than 250 ms were excluded (0.86% of test trials and 0.39% of filler trials)^40^.

#### Response accuracy

Multilevel logistic regression analysis was conducted using R 4.1.1^36^ with packages lme4^41^ and lmerTest^42^; response accuracy was the dependent variable (correct = 1, incorrect = 0). The model included trial type (IN vs. FB trials) as the predictor of fixed effects, specified with random intercepts to accommodate data non-independence of a within-subjects experiment. The random slope of trial type was insignificant, ∆*χ*^2^(2) = 5.28, *p* = 0.071, and was excluded.

Results are shown in Tables 1 and 2. A significant main effect of trial type was observed, *b* = 0.58, *SE* = 0.23, *z* = 2.53, *p* = 0.011, indicating higher accuracy for FB than IN trials. The hypothesis that dual-identity intensionality is more cognitively demanding than false belief in adults was supported.

**Table 1:** Response accuracy in Experiment 1.

Trial type	N	Range	*M* (%)	*SD* (%)
IN	35	0% to 100%	77.14	28.34
FB	35	0% to 100%	82.57	24.42
Fillers	35	18% to 98%	63.21	21.44

**Table 2:** Parameters estimates of mixed model in Experiment 1.

Predictors	Estimates	Odds ratio	Correctness on each trial			
			SE	95% CI^+^	z	p
(Intercept)	1.81	6.13	0.37	[1.09–2.54]	4.92	< 0.001*
Trial type	0.58	1.78	0.23	[0.13–1.02]	2.53	0.011*
Random effects						
σ^2^		3.29				
τ_00_		3.52				
ICC		0.52				
N		35				
Observations		697				
Marginal R^2^/conditional R^2^		0.012/0.523				

*Significant at $$\alpha =0.05$$.

^+^Wald confidence intervals.

#### Response time

A multilevel model analysis specified with random intercepts was conducted to analyse response times from the correct trials. Parameters were estimated using Restricted Maximum Likelihood (REML). The insignificant random slope component, ∆*χ*^2^(2) = 4.29, *p* = 0.117, was not included. Exclusion of the random slope prevented a singular fit with a perfect negative correlation (*r* =  − 1.00) between the random components. There was no significant main effect of trial type, *b* =  − 1.40, *SE* = 47.20, *t*(528.54) =  − 0.03, *p* = 0.976. Hence, the reaction time data alone did not support the hypothesis that intensionality is more cognitive demanding than false belief.

Results of Experiment 1 showed that adults computed false beliefs more accurately, but not faster, than intensionality. From a speed-accuracy trade-off perspective, overall speaking, the present data suggest that it may be more costly for adults to process intensionality than false belief because accuracies differ significantly with similar response times. The finding that adults are more prone to mistakes when processing beliefs involving intensionality in comparison to false beliefs suggest higher processing cost for intensionality than false belief. Experiment 2 set out to examine why processing cost appears to be higher for intensionality than false belief.

## Experiment 2

According to the mental files theory, the observer has to decide which one of two vicarious files is to be deleted to represent the agent’s lack of access to one of the two identities of a dual-identity object in an intensionality situation. In contrast, there is only one vicarious file or identity available to represent the agent’s false thought in the case of identity false belief. Hence, the increased processing cost for intensionality demonstrated in Experiment 1 may be attributed to the availability of more than one identity candidate under the agent’s perspective, compared to false belief. Experiment 2 tested this hypothesis by comparing identities to properties. Each test referent was described as having either alternative identities, as in Experiment 1, or alternative properties. An identity, or sortal concept, represents a set of properties common to a class of referents under a conceptual category; properties are thus embedded in identities^43,44^. In the mental files framework, simpler file management strategies are needed to process descriptions of alternative properties than identities because properties are predicate information stored within a file while identities must be handled by different files. There is no need to create a new file to accommodate an additional property under the same identity. The belief tracker does not need to decide which vicarious file to keep or delete for there is only one vicarious file referring to the object. Therefore, a property version of intensionality should be no more demanding than false belief because they both involve only one identity, or vicarious file, under the agent’s perspective. Lower accuracies and/or longer response times for dual-identity but not property intensionality than false belief would suggest a role of number of vicarious files or identity candidates under the agent’s perspective in determining the processing demand of intensionality versus false belief.

### Methods

#### Participants

Sixty participants were recruited from the Chinese University of Hong Kong via mass email for course credit or a small monetary incentive of approximately USD6.4. They were randomly assigned to the identity (30) and property group (30). Data from 22 of them were discarded due to reports of technical failure (3), i.e., failure to load one or more slides during the experiment, participant ignoring one or more stimulus sentences in any trial (6), and cheating (13), i.e., use of other tools such as paper and pencil to mark down the content of stimulus slides. The remaining thirty-eight participants (20 females) had a mean age of 20.89 years (*SD* = 2.69 years, range = 18 to 32 years); 20 and 18 of them were from the identity and property group, respectively. All participants reported Cantonese–Chinese as their first language, normal hearing, right-handedness, and normal or corrected-to-normal vision. None of them had any special educational needs or colour-blindness. They did not participate in Experiment 1. Power calculation by simulation^37^ showed that test of cross-level interaction effect in a multilevel logistic regression model specified with random intercepts with group sizes 20 and 18 reached 87.80% power, 95% CI [85.61%, 89.76%]. The simulation model assumed a medium effect size (odds ratio 3.47)^38^ and a random intercept variance of 1. Ethical approval was obtained from the Survey and Behavioral Research Ethics Committee, Social Sciences Panel of the Chinese University of Hong Kong. All procedures and methods were performed in strict accordance with the guidelines and regulations set forth by the Committee. All participants gave informed consent before participation.

#### Design and stimuli

Participants assigned to either group (between-subject) had to complete both IN and FB trials (within-subject). Objects were individuated by different identities (nouns) throughout the experiment in the identity group, whereas the description followed as a predicate in the property group. Both groups completed 20 critical test trials all of which required correct rejection, and 80 filler trials, similar to Experiment 1. Response accuracy and response time were recorded as dependent measures.

Stimuli used in the identity group were identical to those in Experiment 1. Each set of stimuli used in the property group included three Chinese adjectives, two of which (A1, A2) were true while the third (A3) was false about the object. Ten sets of adjectives were used in the experimental blocks and an additional ten sets were used in the practice block.

The practice stimuli appeared only in the practice block. The test stimuli sets appeared once in each experimental block. The same set appeared in a different condition in each block and each set appeared ten times in the experiment. Presentation of items in each experimental block and presentation of blocks were randomised. Stimuli were presented through the Gorilla Experiment Builder (www.gorilla.sc)^39^.

#### Procedure

Participants signed on an online consent form before the experiment. Participants in the identity group went through the same procedure as in Experiment 1. For the property group, participants were told that, for example, a tomato was both red (A1) and round (A2), and a scarf could be both long (A1) and soft (A2). Otherwise, procedure was the same as in Experiment 1.

### Results and discussion

Results were pre-screened before analysis. Trials with response times shorter than 250 ms were excluded (1.58% of test trials and 1.12% of filler trials).

#### Response accuracy

Descriptive statistics and model parameter estimates for response accuracy are shown in Tables 3 and 4, respectively.

**Table 3:** Response accuracy in Experiment 2.

Group	N	Trial type	Range	*M* (%)	*SD* (%)
Identity	20	IN	0% to 100%	75.00	29.11
		FB	40% to 100%	91.00	16.19
		Fillers	28% to 86%	67.31	18.68
Property	18	IN	10% to 100%	78.89	30.27
		FB	10% to 100%	83.89	27.68
		Fillers	49% to 91%	70.21	12.99
Overall	38	IN	0% to 100%	76.84	29.33
		FB	10% to 100%	87.63	22.35
		Fillers	28% to 91%	68.68	16.09

**Table 4:** Parameter estimates of mixed model in Experiment 2.

Predictors	Estimates	Odds ratio	Correctness on each trial			
			SE	95% CI^+^	z	p
(Intercept)	3.72	41.34	0.72	[2.31 to 5.14]	5.15	< 0.001*
Description type	− 0.62	0.54	0.90	[− 2.39 to 1.15]	− 0.69	0.492
Trial type	− 1.93	0.15	0.65	[− 3.21 to − 0.65]	− 2.95	0.003*
Description type × trial type	1.13	3.10	0.77	[− 0.38 to 2.64]	1.47	0.141
Random effects						
σ^2^		3.29				
τ_00_		4.76				
τ_11_		1.83				
ρ_01_		− 0.41				
ICC		0.58				
N		38				
Observations		754				
Marginal R^2^/conditional R^2^		0.067/0.605				

*Significant at $$\alpha =0.05$$.

^+^Wald confidence intervals.

Response accuracies were analysed using multilevel logistic regression. A correct response was coded as 1 while an incorrect response was coded as 0. The analysis model included description type (identity group coded as 0, property group coded as 1), trial type (IN trials coded as 1, FB trials coded as 0) and description type × trial type interaction as fixed effect predictors. The random structure included both random intercepts and random slopes of trial type; the effect of random slopes was significant, ∆*χ*^2^(2) = 7.94, *p* = 0.019. Cross-level interaction was insignificant, *b* = 1.13, *SE* = 0.77, *z* = 1.47, *p* = 0.141; description type did not predict response correctness, *b* =  − 0.62, *SE* = 0.90, *z* =  − 0.69, *p* = 0.492. Accuracy for the FB trials was higher than that for the IN trials, *b* =  − 1.93, *SE* = 0.65,* z* =  − 2.95, *p* = 0.003. This result is consistent with the hypothesized greater processing demand for intensionality than false belief. Yet the lack of a description × trial type interaction provides no support for the number of identities account as an explanation for the processing cost difference between intensionality and false belief. Nevertheless, the significant random slope of trial type might suggest methodological flaws that might account for the lack of description × trial type interaction.

To further explore the lack of interaction, separate group analysis was conducted by specifying an intercept- and slopes-as-outcomes model for each group to explore the fixed effect of trial type in each group. It was hypothesised that the fixed effect of trial type would be significant only in the identity but not the property group. The trial type fixed effect in the identity group was significant at $\alpha$ = 0.05, *b* =  − 1.48, *SE* = 0.73, *z* =  − 2.00, *p* = 0.045, but not at the Bonferroni-corrected $\alpha$ = 0.025 level, suggesting a noticeable trend that the IN trials were more difficult than the FB trials for the identity group. In contrast, the trial type fixed effect for the property group was insignificant, *b* =  − 1.10, *SE* = 0.83, *z* =  − 1.33, *p* = 0.183. Hence, separate group analysis indicates a trend that is in line with the hypothesis, which should not be overinterpreted because the cross-level interaction term was insignificant.

It was also noted that accuracies were particularly low in trials in which non-mental verbs were used instead of mental verbs. We suspected that participants might not be fully attending to all the trial content all the time, which might have contributed to the lack of some results.

#### Response time

Response times from the correct trials were analysed by multilevel modelling. Description type, trial type and description type x trial type interaction were entered as predictors of fixed effects. The random structure included random intercepts but excluded the random slope, which was insignificant, ∆*χ*^2^(2) = 0.93, *p* = 0.628, to prevent a singular fit with correlation between random intercept and random slopes being 1.00.

Model results were estimated with REML. Both the main effects of description type, *t*(42.67) = 0.812, *p* = 0.422, and trial type, *t*(594.77) = 0.50, *p* = 0.619, were insignificant. Cross-level interaction was insignificant too, *t*(590.93) =  − 1.49, *p* = 0.137. As in Experiment 1, the present response time data do not support the hypotheses.

## Experiment 3

Experiment 3 was a modification of Experiment 2 designed to answer the same questions. Measures were taken to minimise the possibility of participants adopting non-mentalisitic strategies to solve the trials. The hypotheses were the same as in Experiment 2.

### Methods

Design of Experiment 3 followed that of Experiment 2 with some modifications.

#### Participants

One-hundred-sixty-four participants were recruited via university mass mail, the Chinese University of Hong Kong, and each participant was given approximately USD6.4 as incentive, randomly assigned to the identity (83) and property group (81). After the experiment some of them were excluded due to reports of technical problems (9), reports of consistently ignoring one or more sentences in each trial (2), and cheating behaviour (26). Three participants were excluded because of a combination of the above reasons. Two further participants were excluded due to participation in the previous experiments.

The sample used for further analysis thus included 122 participants (81 females) aged 18 to 57 years (*M* = 22.31 years, *SD* = 5.73 years), 58 and 64 from the identity and property group, respectively. All of them reported Cantonese as their first language and right-handedness. None of them reported any special educational needs nor colour-blindness. They had normal or corrected-to-normal vision and normal hearing. None had participated in Experiment 1 or 2. Forty-eight participants reported not having noticed any change of verbs in the stimulus sentences or failed to recognize the differences between the verbs. This could be due to inattentiveness, as a Mann–Whitney’s U test showed that these participants were less accurate on the non-mental-verb filler trials than participants who attended to the verb differences, *z* = 4.56, *p* < 0.001. If these participants did not attend to the differences between the verbs, we could not rule out the possibility that they adopted a non-mentalistic matching strategy throughout the experiment. They were hence excluded from the main analysis. Hence, the final data set included 74 participants (52 females) aged between 18 and 34 years (*M* = 21.76 years, *SD* = 3.25 years). Ethical approval was obtained from the Survey and Behavioral Research Ethics Committee, Social Sciences Panel of the Chinese University of Hong Kong. All procedures and methods were performed in strict accordance with the guidelines and regulations set forth by the Committee. All participants gave informed consent before participation.

Thirty-five participants were randomly assigned to the identity group and 39 to the property group. Power calculation by simulation^37^ showed that test of cross-level interaction effect in a multilevel logistic regression model specified with random intercepts with group sizes 35 and 39 reached 85.30% power, 95% CI [82.95%, 87.44%], assuming a medium effect size (odds ratio = 3.47)^38^ and a random intercept variance of 2.

#### Stimuli

The stimuli used in Experiment 3 were identical to those used in Experiment 2. Stimulus presentation was controlled by the Gorilla Experiment Builder (www.gorilla.sc)^39^.

#### Procedure

**Figure 4:**
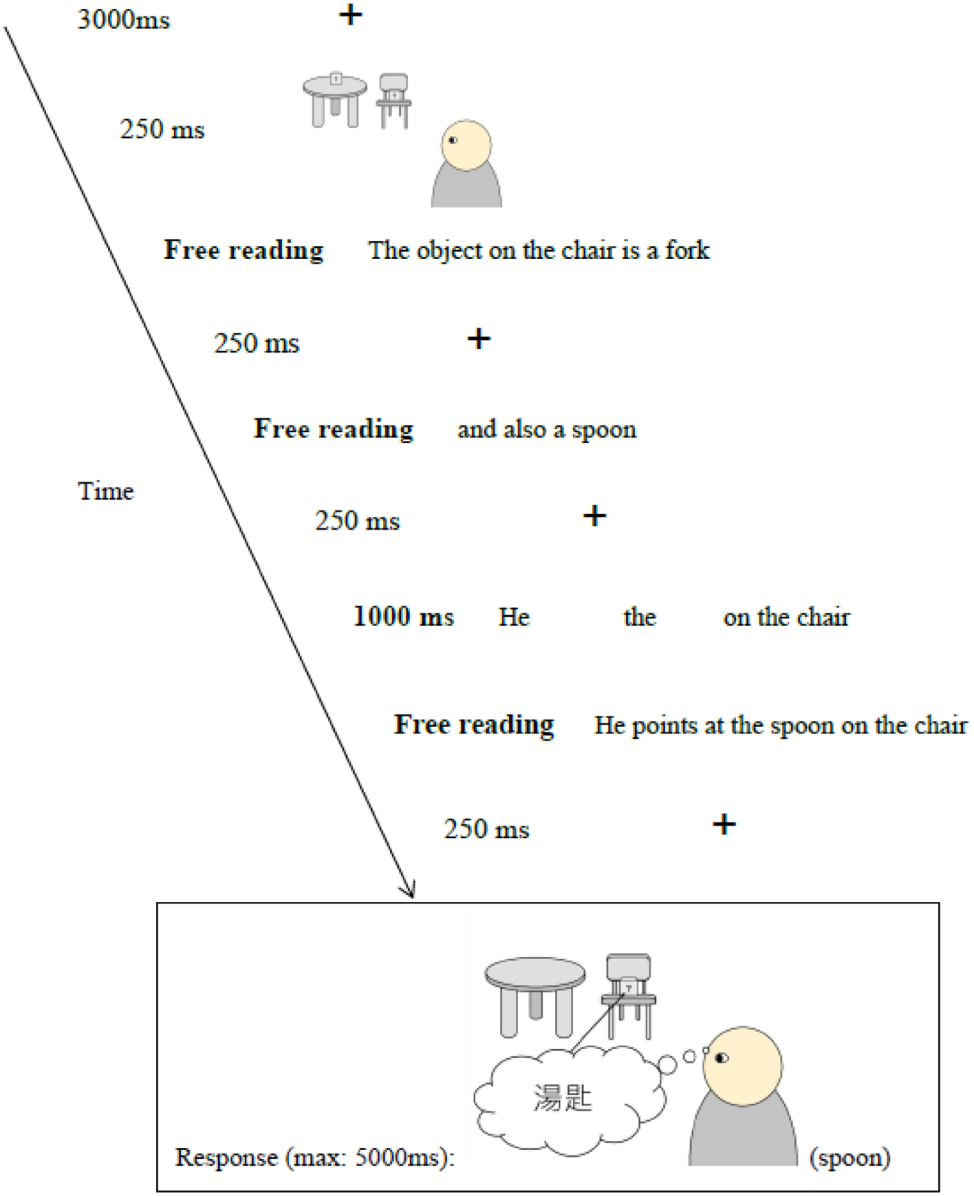
Exemplar of a non-mental-verb filler trial in Experiment 3.

All experiment sessions were conducted in Cantonese–Chinese. Participants signed an online informed consent form before the experiment. The procedure was identical to that of Experiment 2 except for a few modifications, which are described below. Participants were asked to put on headphones through which pre-recorded verbal instruction played automatically when the experiment started. Written instruction was also presented on the computer screen. The verbal instruction recording could not be skipped. To get the participants better prepared for each trial, duration of the fixation cross before each trial was lengthened from 1000 to 3000 ms. An image of the room setting featuring a table with an object on it, a chair with an object in it, and a person agent was briefly presented for 300 ms after the fixation cross. Another fixation cross was then presented for 250 ms before slide 1. On slide 3, a 1000 ms latency of verb and object identity/property presentation was added to make the mental or non-mental verb more salient. Figure 4 depicts the structure of a non-mental-verb filler trial, which required a “no” response. As manipulation check, participants were asked whether they had noticed the change of verb during the experiment and to explain what they thought was the difference between the two verbs in a post-experiment questionnaire.

### Results and discussion

Trials with response times shorter than 250 ms were excluded (2.34% of test trials and 2.55% of filler trials).

#### Response accuracy

Tables 5 and 6 respectively show the descriptive statistics and model parameter estimates for Experiment 3.

**Table 5:** Response accuracy in Experiment 3.

Group	N	Trial type	Range	*M* (%)	*SD* (%)
Identity	35	IN	0% to 100%	78.00	30.56
		FB	30% to 100%	90.57	18.46
		Fillers	34% to 100%	75.50	18.66
Property	39	IN	30% to 100%	90.26	18.99
		FB	10% to 100%	88.46	24.12
		Fillers	40% to 99%	79.13	14.85
Overall	74	IN	0% to 100%	84.46	25.70
		FB	10% to 100%	89.46	21.51
		Fillers	34% to 100%	77.42	16.74

**Table 6:** Parameter estimates of the mixed model in Experiment 3.

Predictors	Correctness on each trial					
	Estimates	Odds ratios	SE	95% CI^+^	z	p
(Intercept)	3.98	53.36	0.53	[2.94 to 5.02]	7.50	< 0.001*
Description type	− 0.41	0.67	0.68	[− 1.73 to 0.92]	− 0.60	0.550
Trial type	− 1.61	0.20	0.30	[− 2.20 to − 1.01]	− 5.29	< 0.001*
Description type × trial type	1.93	6.91	0.43	[1.09 to 2.78]	4.47	< 0.001*
Random effects						
σ^2^	3.29					
τ_00_	5.14					
ICC	0.61					
N	74					
Observations	1473					
Marginal R^2^/conditional R^2^	0.045/0.627					

*Significant at $$\alpha =0.05$$.

^+^Wald confidence intervals.

The multilevel logistic model included description type (identity group coded as 0, property group coded as 1), trial type (IN trials coded as 1, FB trials coded as 0), and description type × trial type interaction as the predictors of fixed effects. Random intercepts were included in the model. Random slopes of trial type were excluded due to insignificance, ∆*χ*^2^(2) = 0.32, *p* = 0.852.

Predicted correctness for the IN trials was significantly lower than that for the FB trials, *b* =  − 1.61, *SE* = 0.30,* z* =  − 5.29, *p* < 0.001; the cross-level interaction was significant, *b* = 1.93, *SE* = 0.43, *z* = 4.47, *p* < 0.001. Description type did not predict correctness, *b* =  − 0.41, *SE* = 0.68, *z* =  − 0.60, *p* = 0.550. Follow-up separate group analyses were conducted by specifying a multilevel logistics model that included random intercepts for each group. Random slopes were excluded due to insignificance, ∆*χ*^2^(2) = 0.33,* p* = 0.850, and ∆*χ*^2^(2) = 1.378, *p* = 0.502, for the identity and property groups, respectively. Correctness for the IN trials was significantly lower than that for the FB trials in the identity group, *b* =  − 1.63, *SE* = 0.31, *z* =  − 5.28, *p* < 0.001, but not in the property group, *b* = 0.32, *SE* = 0.30, *z* = 1.06, *p* = 0.291, using a Bonferroni-adjusted $\alpha$-level of 0.025.

The response accuracy results in Experiment 3 support the hypothesis that the difficulty of processing beliefs that involve dual-identity intensionality, relative to false belief, is explainable by number of identities. Participants in the identity group performed more accurately on the FB than IN trials but the property group did not show this pattern, suggesting that the need to handle more than one identity in intensionality problems may pose additional online processing demand compared to false belief problems. The insignificant random slope component in the main model might indicate that individual differences were better minimised than in Experiment 2, contributing to better sensitivity for the detection of the hypothesised fixed effects.

#### Response time

Response times on correct trials were analysed by multilevel modelling. Predictors of fixed effects were the same as in the response accuracy analysis. Random intercepts were included. Random slopes were excluded due to insignificance, ∆*χ*^2^(2) = 1.54, *p* = 0.463, and the need to avoid a singular fit caused by the perfect correlation of 1.00 between the random intercept and random slopes.

Model results were estimated with REML. The cross-level interaction was insignificant, *t*(1210.19) = 0.05, *SE* = 53.45, *p* = 0.958. The fixed effects of trial type, *t*(1212.86) =  − 0.46, *SE* = 39.54, *p* = 0.648, and description type, *t*(74.55) = 0.66, *SE* = 101.81, *p* = 0.511, were also insignificant. Hence, as in Experiments 1 and 2, the response time data in Experiment 3 did not support the number of identities account. The convergent pattern of results across the three experiments suggests a dissociation between accuracy and response time in non-inferential processing of beliefs.

## General discussion

The three experiments convergently show that adults are more prone to errors when processing beliefs that involve dual-identity intensionality than false beliefs, even when no belief inferencing is involved. Experiment 3 further suggests that number of available identity candidates under the agent’s perspective is important in determining processing demand, as only the identity group, not the property group, made more errors on the intensionality than false belief trials. Describing an object in terms of its distinct alternative identities, which are sortal concepts, triggers conceptual perspective differences on top of mental perspective differences. On the other hand, describing objects as having different properties does not necessitate such conceptual differences. With reference to the mental files theory, this is because sortals are labels of mental files while properties are predicate information stored in mental files. Infant research has suggested that representations of sortal concepts are differentiated from representations of properties at a young age, as 12-month-olds can make use of representations of sortal concepts but not properties to individuate objects in looking-time experiments^45^. Sortal concepts seem to play a more basic role in the individuation of objects.

Nevertheless, response time data in the current experiments do not support the hypotheses. Possibly there is dissociation between response time and accuracy in non-inferential processing of beliefs involving intensionality versus false beliefs. Past literature on learning has also reported such dissociation in humans and monkeys^46–49^ and suggested that the response time/accuracy dissociation may be due to separate mechanisms of learning, such as learning of explicit versus implicit knowledge and materials presented through different modalities. Although the mental files theory is useful for theorising how one manages mental information from different perspectives, the framework is still under development and is not without limitation. Existing empirical works on mental file management focus on explaining ToM development in children by imposing multiple developmental assumptions on file management^18,19,23^, but few, if not none, discuss how adults make mistakes in ToM tasks. Moreover, despite that the framework explains elegantly how false beliefs about object location are traced by distinguishing file content from the files themselves, file management processes involved in keeping track of false beliefs about identity in an appearance-identity task have not been discussed in the literature. The theory suggests that mental files are differentiated by their file labels, which represent sortal concepts, but it remains obscure whether a regular file should be deleted as the person realises that the original label that describes the deceptive appearance rather than the true identity of the object is wrong, or simply have its label updated. This further affects how the vicarious files pointing to the same object are created and deleted. In other words, although the mental files theory makes an effort to spell out what mental operations are involved in handling multiple conceptual and viewpoint perspectives indexed to different agents, many of these operations are still unclear and warrant further empirical investigation. The proposed file management strategies are complicated and may be subject to criticisms on lack of parsimony. More research work is required to further pinpoint the exact mechanism that is critical to the file management strategies specified in the theory.

The current study has the following limitations. First, the dual identities of the items used in the identity condition were arbitrary and the context of the experiment was artificial. This limitation turns out to be a necessary evil as when multiple trials in each condition were conducted to measure the responses of adults, the number of items required was large but there are few “natural” dual-identity objects in reality, not to mention that different people may hold different views on whether a “natural” dual-identity object really possesses both identities or whether the two identities are equally salient. For example, some people may agree that a tomato is a fruit but disagree that it is a vegetable. As detailed in the method section of Experiment 1, we also explained how the current stimulus setup allowed for measures taken to ensure similar semantic associations among the dual identities and the false identity of each target object, thereby ruling out alternative explanations for our results. Hence, setting up the dual identities artificially instead of using “natural” dual-identity objects provides a way to study adults’ processing of beliefs involving intensionality without compromising too much the validity of the current study.

Second, there might not be enough practice before the test trials. As reported in the discussion sections of Experiments 2 and 3, a number of participants failed to acknowledge the difference between the non-mental and mental verbs presented despite the explicit warning in the instruction. More practice trials with feedback could have facilitated participants’ understanding of the instruction. Third, the current study only focuses on simple judgment on belief information, while a more comprehensive model of ToM involves inference, storage and use of information about mental states^14,25^. The current findings may not be generalisable to other stages of ToM processing.

The current experiments focus on adults’ remembering rather than use of belief information or inference of belief. Examining the other stages of processing beliefs that involve intensionality in adults will provide a bigger picture of adult’s ToM processing. Factors affecting reaction times for correct trials in the two trial conditions can also be studied in future research. It is possible that participants may not differ in response accuracy if they are instructed to answer as accurately as possible. Reaction time differences between different carefully designed experimental conditions may inform us more about the exact mental processes underlying processing false beliefs versus intensionality. Finally, future research can probe into the relation between accuracy and reaction time in belief processing. Future experiments could examine the effects on reaction times for correct trials by directly manipulating the processes involved in mental file management while keeping the belief type constant to study whether the proposal of dissociated mechanisms behind management of mental files is supported.

## Data Availability

The datasets generated and analysed during the current study are available in the Open Science Framework repository, http://osf.io/hzpy5/?view_only=b877227a03c145d18f3b977d9e62b94d.
